# Cytokine profiles in the peripheral blood and aqueous humor of patients with herpetic uveitis

**DOI:** 10.1186/s12348-020-00229-9

**Published:** 2020-12-18

**Authors:** Marta Catarina Esteves Guedes, Catarina Gregório Martins, Maria Jorge Arroz, Miguel Angelo-Dias, Luís Miguel Borrego, Rui Daniel Proença

**Affiliations:** 1 Ophthalmology Department, Western Lisbon Hospital Center- Egas Moniz Hospital, Rua da Junqueira, 126, 1349-019 Lisbon, Portugal; 2 CEDOC, NOVA Medical School, Nova University of Lisbon, Campo dos Mártires da Pátria, 1169-056 Lisbon, Portugal; 3 Comprehensive Health Research Centre (CHRC), NOVA Medical School, Nova University of Lisbon, Campo dos Mártires da Pátria, 1169-056 Lisbon, Portugal; 4 Western Lisbon Hospital Center- São Francisco Xavier Hospital, Estr. Forte do Alto Duque, 1449-005 Lisbon, Portugal; 5 Faculty of Medicine, Coimbra Hospital and Universitary Center, Praceta Professor Mota Pinto, 3004-561 Coimbra, Portugal

## Introduction

Uveitis is an intraocular inflammation with several infectious or non-infectious etiologies. The characterization of local and systemic immune profiles in infectious viral uveitis could help recognize different clinical entities and contribute to a more targeted treatment approach.

One previous study addressing cytokine and chemokine profiles in aqueous humor (AqH) of infectious and non-infectious uveitis patients found that interleukin (IL)-1β and IL-10 levels were increased in viral uveitis whereas IL-17 was elevated in toxoplasmic uveitis [1]. IL-10 is of particular interest when studying cytokine profiles in viral infections since it has been shown to suppress the host cellular immune response and favor viral replication, increasing the susceptibility to infection [2] and viral persistence [3].

In this study, we aimed to characterize cytokine profiles in the peripheral blood and AqH of patients with herpetic uveitis (HU) and compare them with healthy controls.

## Methods

### Patients

For this study, both patients and controls were recruited from the Ophthalmology Department of Egas Moniz Hospital, West Lisbon Hospital Center, between October 2014 and October 2016.

Patients presenting with active uveitis from a presumed viral/herpetic etiology were included in the uveitis group. The diagnosis of active uveitis followed the clinical criteria based on inflammatory cell reaction in the anterior chamber or vitreous as per standardization of uveitis nomenclature (SUN) and National Eye Institute (NEI) grading systems [4].

At the time of sampling, all patients had active disease and both blood and AqH samples were collected at presentation. Intraocular samples were examined for the presence of cytomegalovirus (CMV), herpes simplex virus (HSV)-1 and 2, and varicella zoster virus (VZV) by real-time polymerase chain reaction (PCR) analysis as previously described [5].

Controls were selected among healthy subjects undergoing cataract or refractive surgery, with no known history of intraocular inflammation.

The study protocol was approved by the Ethics Committee of Egas Moniz Hospital, West Lisbon Hospital Center, and informed consent was obtained from each patient.

### Sample collection

The AqH samples were collected with a 30-gauge needle under topical anesthesia and sterile conditions by slit lamp with the aid of one drop of povidone iodine before and after puncturing the anterior chamber. The AqH samples of control subjects were collected with a 30-gauge needle before starting surgery. Undiluted aqueous samples of at least 0.1 mL were collected from each subject and immediately sent to the laboratory for analysis.

Peripheral blood samples were also collected in order to obtain serum.

### Quantification of serum cytokine expression by multiplexed flow cytometry

A multiplex bead-based immunoassay (BD CBA Flex Set, BD Biosciences, San Jose, CA, USA) was used to determine serum and AqH levels of TNF-α, IFN-ɣ, IL-17A and IL-10. A similar single-plex bead-based immunoassay was used for TGF-β.

The protocol was performed following the instructions of the manufacturer. In brief, standards and serum samples were incubated with specific capture beads for 1 h at room temperature. After adding the detection reagent, the mixtures were incubated for 2 h at room temperature in the dark. After a final wash, beads were acquired in a BD FACS Canto II, previously set up for the BD CBA Flex Set. For each cytokine, at least 300 beads were acquired per sample. The FCAP Array Software (BD Biosciences) was used for data analysis. Standard curves covered a 0–2500 pg/mL concentration range and the minimum detection levels were: 0.13 pg/mL for IL10; 0.3 pg/mL for IL17A; 1.8 pg/mL for IFN-γ and 0.7 pg/mL for TNF-α.

For TGF-β, analyzed separately, samples were previously activated with the Sample Activation Kit 1 (R&D, Minneapolis, MN, USA) according to the recommended procedure. After activation, samples were incubated with capture beads for 2 h, washed and incubated with detection reagent. Acquisition and analysis were performed as described above. For TGF-β, standard curves covered a 0–10,000 pg/mL concentration range, and minimum detection level was 14.9 pg/mL.

### Statistical analysis

The Mann-Whitney *U* test was used to compare each 2 independent groups. A *P* value of < 0.05 was considered for statistical significance. Data were analyzed using GraphPad Prism, version 8 for Windows (GraphPad Software, La Jolla, California).

## Results

Four patients with presumed HU and 8 controls were included. Table 1 summarizes the demographic and clinical features for the HU group. One patient with a panuveitis associated with acute retinal necrosis was also included. All HU patients underwent anterior chamber puncture and AqH sampling as previously described. Results of AqH by real-time PCR confirmed a VZV infection in all the cases tested.

**Table 1:** Demographic and clinical features for the HU group

Patient	Gender	Age (years)	Clinical features at presentation	Sytemic features	PCR results
1	F	76	Unilateral anterior uveitis; sectorial iris atrophy; diffuse KPs; elevated IOP	None	+ VZV
2	M	45	Panuveitis- acute retinal necrosis	None	+ VZV
3	F	84	Unilateral queratouveitis; sectorial iris atrophy; diffuse KPs	None	+VZV
4	M	74	Unilateral anterior uveitis; elevated IOP; cataract; diffuse KPs; diffuse iris atrophy	None	+VZV

*F* female, *M* male, *KPs* keratic precipitates, *IOP* intraocular pressure, *VZV* varicella zoster virus, *PCR* polymerase chain reaction

Regarding serum cytokines, there were no significant differences observed between patients and controls.

In AqH samples however, patients showed increased concentrations of IL10 (*p* = 0.018), TNF-α (*p* = 0.018) and IFN-ɣ (*p* = 0.024).

Interestingly, the levels of serum and AqH cytokines differ within the two groups. While controls presented higher levels of IL10, IFN-ɣ and TGF-β in serum samples compared to those found in AqH (respectively, *p* = 0.001; *p* = 0.002 and *p* = 0.001), in the patients’ group, only TGF-β showed higher serum concentrations when compared to AqH (*p* = 0.029), with comparable values for the other cytokines tested.

Table 2 shows the results and comparison of cytokine levels in the serum and AqH of both groups.

**Table 2:** Comparison of cytokine levels in HU patients and controls

Serum & aqueous humor (AqH) cytokines	Controls, serum samples (***n*** = 8)	Controls, AqH samples (***n*** = 8)	HU, serum samples (***n*** = 4)	HU, AqH samples (***n*** = 4)	Controls vs HU, serum samples	Controls vs HU, AqH samples	Controls, Serum vs AqH samples	HU, serum vs AqH samples
**pg/ml, median (IQR)**								
IL-10	1,22 (0,42-1,69)	0,00 (0,00-0,00)	1,88	3,44	–	**0.018**	**0.001**	**–**
IL-17A	1,20 (0,00-3,73)	1,20 (0,00-3,73)	0,20	0,99	**–**	**–**	**–**	**–**
TNF-α	0,00 (0,00-0,82)	0,00 (0,00-0,00)	0,18	0,91	–	**0.018**	–	**–**
INF-ɣ	2,81 (1,58-4,83)	0,00 (0,00-0,05)	2,08	0,87	**–**	**0.024**	**0.002**	**–**
TGF-β	1962 (1685–2356)	368 (0,00–565)	1861	374	**–**	–	**0.001**	**0.029**

*HU* Herpetic Uveitis, *IQR* interquartile range, *AqH* Aqueous humour

Mann-Whitney nonparametric *U* test was used for group’s comparison. Results are presented as medians and interquartile range, median (IQR). Statistically significant results are indicated in bold

## Discussion

Despite including just a few HU cases, our preliminary results show an elevation of intraocular TNF-α and IFN-ɣ levels which is likely associated with active disease and anterior chamber inflammation.

The increase in IL-10 levels also found in patients’ AqH samples is, as described in a previous study [1], probably related to the viral etiology, in this case herpetic. However, since all the patients included tested positive for VZV infection, it is possible that other herpetic etiologies, such as HSV-1 and 2 or CMV, show different serum and intraocular cytokine profiles.

The immunosuppressive ability of IL-10 to impair T-cell responses leading to persistent viral infection has already been demonstrated in mice [6, 7], reinforcing its possible use as a biomarker for viral infection or even as a therapeutic target since it has been shown that in vivo administration of an antibody against the murine IL-10 receptor completely prevented the viral persistence by restoring T-cell function [3].

In conclusion, although more studies are needed to confirm our findings, elevated intraocular IL-10, TNF-α and IFN-ɣ levels seem to be associated to VZV-associated uveitis.

## Data Availability

The datasets used and/or analyzed during the current study are available from the corresponding author on reasonable request.

## Abbreviations

AqH: Aqueous humor
CMV: Cytomegalovirus
HSV: Herpes simplex virus
HU: Herpetic uveitis
IFN-ɣ: Interferon-Ɣ
IL: Interleukin
PCR: Polymerase chain reaction
TGF-β: Transforming Growth Factor β
TNF-α: Tumor Necrosis Factor α
VZV: Varicella zoster virus
